# Assessment of knowledge, attitude, and practice of dog owners to rabies disease in Kahama town council, Shinyanga region, Tanzania

**DOI:** 10.1371/journal.pntd.0011580

**Published:** 2023-09-06

**Authors:** Shabani Iddi, Farida Mlenga, Kayo Hamasaki, Stanley Mwita, Eveline Konje

**Affiliations:** 1 Department of Physiology, Catholic University of Health and Allied Sciences, Mwanza, Tanzania; 2 Department of Pharmaceutics and Pharmacy Practice, Catholic University of Health and Allied Sciences, Mwanza, Tanzania; 3 Department of Biostatistics Epidemiology and Behavioral Sciences, Catholic University of Health and Allied Sciences, Mwanza, Tanzania; Colorado State University, UNITED STATES

## Abstract

**Background:**

Rabies is a fatal zoonotic disease of significant public health importance. Domestic dogs are the main reservoir and transmitter of this disease, particularly in developing countries. Community awareness about rabies is one of the key components of prevention. This study describes the knowledge, attitudes, and practices about rabies disease among dog owners at Kahama town council, Shinyanga Region, Tanzania.

**Methodology:**

This was a cross sectional community-based study which was done in May 2021. Structured questionnaires were administered to collect the data among 422 dog owners. The information collected included demographic characteristics of the dog owners, as well as their knowledge, attitude and practice towards rabies. Data were analyzed using SPSS statistical software version 20.

**Results:**

Out of 422 respondents, 421 (99.76%) knew that rabies can be transmitted by dogs, 384 (91%) knew that rabies can be prevented by vaccination of dogs, 269 (63.74%) knew the symptoms and signs, and 379 (89.81%) believed that it was necessary to vaccinate all owned dogs, but 227 (53.79%) had not vaccinated their dogs. Education level (p = 0.006) and occupation (p = 0.002) were significantly associated with a positive attitude, whereby those with a higher level of education and farmers were more likely to have a positive attitude. Also there was statistically significant association between gender (p = 0.038), marital status (p < 0.001) occupation (p < 0.001), education level (p = 0.006) and the practices of the respondents in the community whereby majority of male, unmarried dog owner who are farmer with primary education level had lower practice score.

**Conclusion:**

This study concludes that respondents had good knowledge, a relatively good attitude, and poor practice towards rabies prevention and control. Rabies awareness with an emphasis on the importance of vaccination as well as vaccination campaigns should, therefore, be intensified, especially among the least educated dog owners.

## Author summary

Rabies is a fatal zoonotic disease of significant public health importance. Domestic dogs are the main reservoir and transmitter of this disease, particularly in developing countries. Community awareness about rabies is one of the key components of prevention. Here we assessed the knowledge, attitudes, and practices about rabies disease among dog owners at Kahama town council, Shinyanga Region, Tanzania. The finding shows that, majority of respondents had good knowledge and relatively good attitude, however a good number of them also had poor practice toward rabies prevention and control. It was further observed that both attitude and practice were associated with education level and occupation. The observed association could be due to the fact that education level and occupation of the respondents have influence in good attitude and practices regarding rabies disease and anti-rabies vaccination including vaccination of their dogs and willingness in paying full costs of post exposure prophylaxis. In conclusion, despite good knowledge and positive attitude about rabies among dog owners in Kahama town council, the practice was found to be poor indicating practice gaps in the community. We recommend that rabies awareness and vaccination campaigns be intensified, particularly among the least educated dog owners. Further, similar studies should be conducted in other regions of the country, especially in communities with a large number of dogs.

## Background

Rabies is a neglected viral zoonotic disease caused by a negative stranded RNA virus from Genus Lyssa virus from the family Rhabdoviridae [1]. Rabies remains a significant cause of about 55,000 deaths each year worldwide, predominantly among children and the rural poor in Asia and Africa [2]. The disease claims 24,000 human deaths annually in Africa alone [3]. Rabies is endemic in Tanzania with an estimated 1500 deaths each year according to World Health Organization (WHO) [4]. Rabies burden in Tanzania was 4.9 human deaths /100,000 and 0.62 human deaths /100,000 based on active surveillance and on national bites statistics respectively [5]. Domestic dogs are considered to be the main source for human rabies in Africa [6]. Although a wide range of animals can become infected and transmit the disease, only mammals from the Carnivora and Chiroptera (bats) order act as the reservoir of the disease [6]. Transmission to people occurs predominantly through infected animal bite or scratch, as well as their saliva through the human mucosa or broken skin. Rabies invades the central nervous system and in absence of post exposure prophylaxis (PEP), it becomes fatal once clinical signs appear [7]. Symptoms can be nonspecific but often includes hydrophobia, hyper salivation, biting, aggression and respiratory difficulties. Anti-rabies vaccine is a vaccine used to prevent rabies before, and for a period of time after, exposure to the rabies virus. The world Health Organization recommended PEP protocol includes immediate wound washing, administration of anti-rabies vaccine and in severe exposures, infiltration of purified rabies immunoglobulin (RIG) into the wounds [8]. Mass dog’s vaccination annually, is a life saving measure to prevent the fatal onset of rabies. To successful eliminate rabies, vaccination must reach at least 70% of the dog population over the consecutive years [1]. Vaccination rates lower than 30% are considered to be a waste of resources [6]. Also, deaths from rabies can be prevented by timely application of PEP. However, rabies incidence is likely to be much higher considering the lack of accurate data and underreporting of cases. Published data of rabies from urban and rural areas of Tanzania, including pastoralists are still lacking. However, despite the efforts of rabies elimination, most vaccination efforts in Africa have failed to achieve high levels of coverage. These interventions are influenced by local dog ownership practices and under compliance of dog vaccination campaign. This brings a necessity for further studies, through involvement of the community to address this problem.

Community awareness about rabies is very important in rabies prevention and control. The knowledge gap among the community should be identified and targeted. The aim of this study is to assess the level of knowledge, attitudes, and practices regarding rabies among household dog owners of Kahama town council, Shinyanga.

## Material and methods

### Ethics statement

This study received ethical approval from the Joint Catholic University of Health and Allied Sciences/Bugando Medical Centre (CUHAS/BMC) Research Ethics and Review Committee with ethical clearance certificate number CREC/1829/2021. Permission to conduct the study was obtained from the Executive Director of Kahama town Council. Written informed consent was obtained from each participant before being recruited to participate in the study. Those who volunteered to participate were assured of confidentiality.

### Study design, area and population

The study was a cross-sectional community-based study conducted among household dog owners aged 18 years and older at Kahama town council in Shinyanga region from 1^st^ May to 30^th^ May 2021. Administratively, the town council’s public administration is made up of one division, namely Kahama town, which has 11 wards, and some of the area from Dakama’s 3 wards, Msalala’s 2 wards, and Isagehe’s 4 wards, totalling 20 wards for the Kahama town council. Kahama town council covers an area of 817,641.1 Ha (817.64 Km^2^). The population of Kahama town council was estimated to be 453,654 people as per census of 2022 [9].

### Sample size and sampling procedure

Sample size was calculated by using Cochran’s sample size formula (1977) for categorical data. [10]. Using the proportion of 50% (the known population) from the study done in Gondar Zuria district, Ethiopia, the minimum sample size obtained was 384. Adding the 10% non-response rate, the final estimated total sample size was 422 subjects (dog owners).

Random sampling procedure was used in a subset of 422 sample size of households which owns dogs. Kahama town council has 20 wards. Only five wards were chosen from the 20 wards using a lottery selection method whereby each ward was assigned a number, after which numbers were selected at random, each ward among the 20 wards had the same probability of being selected. Within the five chosen wards, the total number of households owning dogs was identified through field visits. Owing to the lack of proper sampling frame, a door-to-door survey was conducted using a rolling sample method (in which the first selected household provided information about the next available household in the area or within the village) until the target number of household’s respondents was achieved. From each household all household’s heads or dog owners above 18 years of age who agreed to participate in the study were interviewed.

### Data collection procedure

Data collection was done by the principal researcher and trained research assistants using a structured and pretested questionnaire with questions partly adapted from similar studies conducted elsewhere and consisting of closed and a few open questions.The information collected included demographic characteristics of the dog owners, knowledge, attitude and practice of dog owners towards rabies.

### Data analysis

The data was cleaned, and checked for its completeness and consistency and then corrected if possible or removed if otherwise. The data was coded and entered into Microsoft Excel and then transported to Statistical Package for the Social Sciences (SPSS) version 20 for analysis [11]. Then, the frequency distribution of both dependent and independent variables was sort or worked out by using descriptive statistical techniques such as Mean, Standard deviation, Percentages and frequencies. Lastly, association between independent variables and Knowledge, Attitude and Practices (KAP) scores on rabies disease and ant rabies vaccination were calculated using Pearson’s Chi square or Fisher`s Exact test where appropriate. In all analysis significance level was set at p-value of less than 0.05.

Scores were given according to the completeness and accuracy of respondent’s answers. If all answers were complete and accurate, a respondent obtained overall scores of 12, 15 and 15 respectively for knowledge, attitude and practices. A respondent was considered to have good knowledge, positive attitude or good practices about rabies, if he or she obtained a score of 8 or more out of 11 (for knowledge), 10 or more out of 15 (for attitude) and 10 or more out of 15 (for practices) respectively which is equal to or more than 60% according to the cut-off point of Likert-type scale [12,13]. For those who scored less than 60% for each category were considered to have poor knowledge, negative attitude or poor practices respectively.

## Results

### Socio demographic characteristics of the participants

A total of 422 household dog owners, with a mean age of 38.9 ± 14.2 were involved in the study. There were 249 (59.00%) respondents between the ages of 19 and 39. Of the 422 respondents who participated in the study, 315 (74.64%) were male. The majority of respondents, 301 (71.33%), were Christians; 317 (75.12%) were married; 274 (64.93%) had a primary school education; and 234 (55.4%) were farmers (Table 1).

**Table 1:** Socio-demographic data of the study participants (N = 422).

Variables	Frequency (n)	Percentage (%)
**Ages (years)**		
19–39	249	59.00
40–59	118	27.96
60–79	52	12.32
80–99	3	0.71
**Gender**		
Male	315	74.64
Female	107	25.36
**Religion**		
Christian	301	71.33
Muslim	75	17.77
Pagan	45	10.66
Others	1	0.24
**Marital status**		
Single	88	20.85
Married	317	75.12
Divorced	5	1.18
Widow/Widower	12	2.84
**Education level**		
No formal education	13	3.08
Primary school	274	64.93
Secondary school	99	23.46
College/ University	36	8.53
**Occupation**		
Farmer	234	55.45
Business	119	28.20
Student	2	0.47
Employee	61	14.45
Housewife	5	1.18
Dependent	1	0.24

### Knowledge of the participants about rabies disease and ant rabies vaccination

All 422 respondents (100%) had heard of rabies disease and believed that it is a fatal disease. More than half of the respondents 266 (63.03%) had heard about rabies from friends. Almost all respondents 421 (99.76%) knew that rabies can be transmitted by dogs and majority 269 (63.74%) were aware of the symptoms of a rabid dog. More than three quarter 384 (91%) of respondents knew that rabies can be prevented by vaccination of dogs and more than half of the respondent 239 (56.64%) were aware that rabies can be prevented by giving a post exposure prophylaxis (ant rabies vaccine) to a person bitten by a rabid dog (Table 2).

**Table 2:** Assessment of knowledge on rabies among the study participants.

Knowledge item N = 422	Frequency (n)	Percentage (%)
Have you heard of rabies?		
Yes	422	100
No	0	0
Where did you hear it from?		
Friends	266	63.03
Health center	57	13.51
Magazine	35	8.29
Others	33	7.82
Television	31	7.35
Do you believe that rabies is a fatal disease?		
Yes	422	100
No	0	0
Do you believe that rabies can be transmitted by dogs??		
Yes	421	99.76
No	1	0.24
Do you know the symptoms of a rabid dog?		
Yes	269	63.74
No	153	36.26
Do you believe that rabies can be prevented by vaccination of dogs?		
Yes	384	91.00
No	38	9.00
Do you believe that rabies can be prevented by vaccination of human beings?		
Yes	239	56.64
No	183	43.36
How is rabies disease transmitted?		
Through dog biting		
Yes	421	99.76
No	1	0.24
Do you believe that suspected rabies can be confirmed by laboratory test?		
Yes	305	72.27
No	117	27.73

According to the knowledge scores based on the questions asked, 416 (98.58%) participants had good knowledge and 6 (1.42%) had poor knowledge about rabies disease and anti-rabies vaccination. In multivariate analysis, there was no association of knowledge with any of the socio-demographic characteristics (Table 3).

**Table 3:** Association of demographic characteristics of respondents with categorized knowledge score of rabies among study participants.

Variables	Categorized score (N = 422)		P–value*
	Poor Number (%)	Good Number (%)	
**Age (years)**			1.000
19–39	4 (2.01)	244 (.97.99)	
40–59	2 (1.69)	117 (98.31)	
60–79	0 (0.00)	52 (100.00)	
80–99	0 (0.00)	3 (100.00)	
**Gender**			0.525
Male	5 (1.59)	310 (98.41)	
Female	1 (0.93)	106 (99.07)	
**Religion**			0.206
Christian	4 (1.33)	297 (98.67)	
Muslim	0 (0.00)	74 (100.00)	
Pagan	2 (4.44)	43 (95.56)	
Others	0 (0.00)	1 (100.00)	
**Marital status**			0.737
Single	2 (2.27)	86 (97.73)	
Married	4 (1.58)	313 (98.42)	
Divorced	0 (0.00)	5 (100.00)	
Widow/Widower	0 (0.00)	12 (100.00)	
**Education level**			
No formal education	0 (0.00)	13 (100.00)	0.504
Primary school	6 (2.20)	268 (97.81)	
Secondary school	0 (0.00)	99 (100.00)	
College/University	0 (0.00)	36 (1000.00)	
**Occupation**			
Farmer	6 (2.56)	228 (97.43)	0.435
Business	1 (0.84)	118 (99.16)	
Student	0 (0.00)	2 (100.00)	
Employee	0 (0.00)	61 (100.00)	
Housewife	0 (0.00)	5 (100.00)	
Dependent	0 (0.00)	1 (100.00)	

*Fisher`s Exact

### Attitude of the respondents towards rabies disease and ant rabies vaccination

According to the attitude score, out of 422 respondents, 345 (81.75%) had a positive attitude, while 77 (18.25%) had a negative attitude. Most respondents, 406 (96.21%), said they would go to the hospital for treatment if bitten by a stray dog or by an owned dog, 392 (92.89%). Moreover, 387 (91.71%) reported that they would report to the authorities if there was a suspected rabies outbreak in the community. Almost all of the respondents 411 (97.39%) supported a rabies control campaign in their community, and 379 (89.81%) believed that it was necessary to vaccinate all stray dogs. The majority of respondents, 321 (76.07%), believed that killing dogs is the best way to control the dog population, while 305 (72.27%) said they would kill stray dogs if they suspected rabies disease, and 356 (84.36%) said it is necessary to get post-exposure prophylaxis after a dog bite.

Table 4 presents rabies attitude score among study participants based on sociodemographic characteristics. There was a statistically significant association between the attitude of dog owners and their level of education (p = 0.004) and occupation (p = 0.001). The majority of dog owners with a positive attitude had a primary school education and were farmers.

**Table 4:** Association of demographic characteristics of respondents with categorized attitude score of rabies among study participants.

Variables	Categorized score (N = 422)		P- value
	Negative Number (%)	Positive Number (%)	
**Age (years)**			0.280*
19–39	48 (19.28)	201 (80.72)	
40–59	11 (11.34)	86 (88.66)	
60–79	7 (13.46)	45 (86.54)	
80–99	0(0.00)	3(100.00)	
**Gender**			0.890**
Male	57 (18.10)	258 (81.90)	
Female	20 (18.69)	87 (81.31)	
**Religion**			0.460*
Christian	50 (16.61)	251 (83.39)	
Muslim	17 (22.67)	58 (77.33)	
Pagan	10 (22.22	35 (77.78	
Others	0 (0.00)	1 (100.00)	
**Marital status**			0.225*
Single	20 (22.73)	68 (77.27)	
Married	56 (17.67)	261 (82.33)	
Divorced	1 (20.00)	4 (80.00)	
Widow/Widower	0 (0.00)	12 (100.00)	
**Education level**			
No formal education	2 (15.38)	11 (84.62)	0.004*
Primary school	63 (23.00)	211 (77.00)	
Secondary school	10 (10,10)	89 (89.90)	
College/University	2 (5.56)	34 (94.44)	
**Occupation**			
Farmer	56 (23.93)	178 (76.07)	0.001*
Business	13 (10.92)	106 (89.08)	
Student	0 (0.00)	2 (100.00)	
Employee	5 (8.20)	56 (91.80)	
Housewife	2 (40.00)	3 (60.00)	
Dependent	1 (100.00)	0 (0.00)	

*Fisher`s exact

**Chi square

### Practice of the respondents towards rabies disease and ant rabies vaccination

The majority of study participants, 172 (40.76%), owned one dog, while 54 (12.8%) owned more than three dogs. More than half of respondents, 277 (65.64%), owned dogs to guard their livestock against thieves and wild animals. About 106 (25.12%) of the respondents reported that they would visit the hospital immediately following a dog bite, while 316 (74.88%) reported that they would use other alternatives before reporting immediately to the hospital. Most of the respondents, *227* (53.79%), had not vaccinated their dogs, while the majority of 304 (72.04%) agreed that they leave their dogs to roam around the streets (Table 5).

**Table 5:** Assessment of practice towards rabies among the study participants.

Practice item	Frequency (n) N = 422	Percentage (%)
**Number of dogs owned**		
One	172	40.76
Two	131	31.04
Three	65	15.40
>Three	54	12.80
**Reason for keeping dog**		
Hobby	28	6.64
Guarding the house	117	27.73
Guarding livestock	277	65.64
**Does your dog free roam around the streets?**		
Yes	304	72.04
No	118	27.96
**Does your dog eat around the streets?**		
Yes	117	27.78
No	305	72.22
**When exposed to rabies, do you use traditional treatment?**		
Yes	292	69.19
No	130	30.81
**Do you vaccinate your dogs?**		
Yes	195	46.21
No	227	53.79
**Would you get a post exposure prophylaxis vaccine after a dog bite?**		
Yes	116	27.49
No	306	72.51
**Do/would you visit the hospital as soon as you are exposed to a dog bite?**		
Yes	106	25.12
No	316	74.88
**Do you educate other community members on the importance of ant rabies vaccination?**		
Yes	158	37.44
No	264	62.56

The majority of the respondents, 324 (76.78%), had poor practices about the rabies disease and anti-rabies vaccination, and only 98 (23.22%) had good practices. Table 6 presents scores on rabies practices by sociodemographic characteristics among study participants. There was a statistically significant association between the practice of dog owners and their gender (p = 0.038), marital status (p < 0.001), occupation (p < 0.001) and level of education (p = 0.006). The majority of dog owners with poor practices were male, single, famer and had a primary school education.

**Table 6:** Association of demographic characteristics of respondents with categorized practices score of rabies among study participants.

Variables	Categorized score (N = 422)		P–value
	Poor Number (%)	Good Number (%)	
**Age (years)**			0.576*
19–39	192 (77.11)	57 (22.89)	
40–59	93 (78.81)	25 (22.19)	
6079	37 (71.15)	15 (28.85)	
80–99	2 (50.00)	1 (50.00)	
**Gender**			0.038**
Male	234 (74.29)	81 (25.71)	
Female	90 (84.11)	17 (15.89)	
**Religion**			0.909*
Christian	230 (76.41)	71 (23.59)	
Muslim	57 (76.00)	18 (24.00)	
Pagan	36 (80.00)	9 (20.00)	
Others	1 (100.00)	0 (0.00)	
**Marital status**			< 0.001*
Single	243 (92.05)	21 (7.95)	
Married	67 (47.52)	74 (52.48)	
Divorced	5 (100.00)	0 (0.00)	
Widow/Widower	9 (75.00)	3 (25.00)	
**Education level**			0.006*
No formal education	11 (84.62)	2 (15.38)	
Primary school	226 (82.48)	48 (17.52)	
Secondary school	65 (65.66)	34 (34.34)	
College/ University	11 (84.62)	2 (15.38)	
**Occupation**			< 0.001*?
Farmer	200 (85.47)	34 (14.53)	
Business	79 (66.39)	40 (33.61)	
Student	1 (50.00)	1 (50.00)	
Employee	40 (65.57)	21 (34.43)	
Housewife	3 (60.00)	2 (40.00)	
Dependent	1 (100.00)	0 (0.00)	

*Fisher`s exact

**Chi square

## Discussion

According to the findings of this study, the respondents of Kahama town council in Shinyanga had a good level of knowledge (98.58%) and attitude (81.75%) toward rabies disease and anti-rabies vaccination. The good level of knowledge among the respondents may be due to the endemicity of rabies and frequent reports of rabies outbreaks in their communities. This corresponds to previous studies in Gujarat, India [14] and Gondar, Zuria, Ethiopia [15] which reported good knowledge in 100% and 98% of respondents respectively. However, the percentage of respondents with good knowledge is higher than in other reports from Addis Ababa, Ethiopia [16], and India [17] that reported 83% and 68.7%, respectively. The reason for the discrepancy could be due to real differences in the study areas. In our study, the respondents were heads of households or dog owners living in rural and urban areas. This group is likely to have better communication and information about what is happening in their residential areas, including animal disease situations, which can be through mass media, friends, mass dog vaccination campaign and information that they might get when taking their dog for veterinary services such as vaccination and treatment, and therefore may contribute to their good knowledge.

The attitudes of the study respondents were also good, whereby majority of the respondents, 345 (81.75%), had a positive attitude towards rabies prevention and control. More than three-quarters of respondents thought that it was necessary to get post-exposure prophylaxis after a dog bite, and more than 90% said they would go to the hospital if bitten by a stray or their own dog. This corresponds to a study done in Gelephu, south central Bhutan [18].

Despite a good level of knowledge and a positive attitude about rabies among the respondents in the current study, practice was found to be poor. Respondent’s poor practice especially that of not reporting immediately to the hospital after a dog bite, could be contributed by poverty, traditional beliefs, the high cost of PEP, its unavailability, and difficulties in accessing PEP at the nearby health centers, especially in remote rural areas due to shortages of PEP in health facilities [19–23]. It is difficult for bite victims in rural areas to reach the major health facilities where PEP can be obtained due to long distance and poor transportation than in urban areas [21]. Also patients who needed to be escorted by a family member or adult had their indirect costs more than doubled, with rural bite victims incurring higher indirect costs and a higher risk of developing a fatal disease. Failure to initiate PEP or delays in receiving PEP due to hospital shortages and costs (both direct and indirect) may deter poor people living in remote rural areas from obtaining and completing PEP [20,24,25]. Also, the distance to major hospitals seems to be an obstacle for people living in rural areas in Africa [21]. A study in Ivory Coast [26] reported that over 75% of patients who discontinued PEP were from outside the capital city, Abidjan. In summary, this study has shown that major inequalities in health care and access to and affordability of PEP for bite victims also exist in Tanzania and has demonstrated the importance of evaluating health-seeking behavior in local settings.

There was statistically significant association between gender (p = 0.038), marital status (p < 0.001) occupation (p < 0.001), education level (p = 0.006) and the practices of the respondents in the community whereby majority of male dog owner who are not married (single) and farmer with primary education level had lower practice score. Also, there was statistically significant association between education (p = 0.004) and attitude of respondents and between occupation (p = 0.001) and the attitude of respondents. This corresponds to the finding of the previous studies [4,13,16,27,28]. The observed association could be due to the fact that education level and occupation of the respondents have influence in good attitude and practices regarding rabies disease and anti-rabies vaccination which includes vaccination of their dogs and willingness in paying full costs of PEP.

### Study limitations

This study being limited to Kahama town council, the findings may not be generalizable to Shinyanga region and Tanzania as a whole, but they did provide data for Kahama district, one of the highly populated district of Shinyanga region. Also we did not inspect the vaccination certificates of dog owners as this could verify the self-report they gave regarding their vaccination of dogs and added more value to the study. Further, the study is limited by absence of serology results as presence of serology data alongside reported vaccination status of dogs from the area can effectively reduce bias, enhance overall quality and bridge the knowledge gap.

### Conclusion

Despite good knowledge (98.58%) about rabies and a positive attitude (81.75%) towards rabies prevention and control among dog owners in Kahama town council, the practice was found to be poor (76.78%) indicating practice gaps in the community. We recommend that rabies awareness and vaccination campaigns be intensified, particularly among the least educated dog owners. Further, similar studies should be conducted in other regions of the country, especially in communities with a large number of dogs.
